# Supplementary material for: PGC1/PPAR drive cardiomyocyte maturation at single cell level via YAP1 and SF3B2
Source: Nat Commun. 2021 Mar 12;12:1648. doi: 10.1038/s41467-021-21957-z (PMC7955035; doi:10.1038/s41467-021-21957-z)
Supplement: Supplementary file 1 — Supplementary Information [file 41467_2021_21957_MOESM1_ESM.pdf]

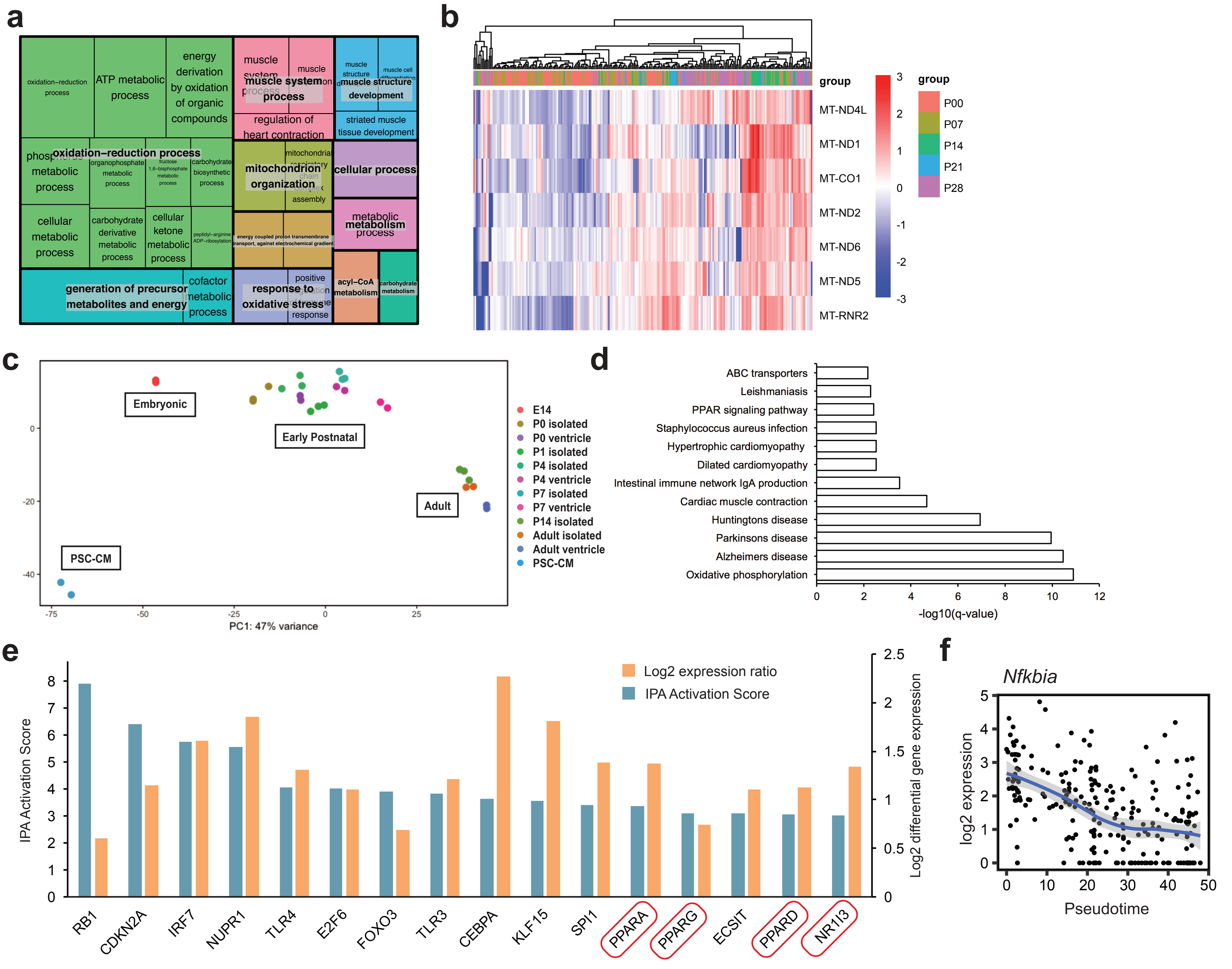

Fig. S1: scRNA-seq clustering and meta-analysis of bulk RNA-seq
a, Treemap of GO terms of differentially expressed genes from P0 to P28 with box size representing the -log10 p-value. **b,** Heatmap of mitochondrial gene expression with hierarchical clustering of time-points. **c,** PCA plot of two components of bulk RNA-seq datasets of cardiac maturation (GSE64403, GSE47948, GSE95762, GSE79883). **d,** KEGG pathway mapping of differentially expressed genes from neonatal to adult CMs. **e,** Top IPA transcriptional regulators with log fold change (orange) and IPA activation score (blue) visualized. PPAR family nuclear receptors are among the top hits.
f, Expression trend over pseudotime of NFκBIα in postnatal CMs. Interval represents 95% confidence interval.

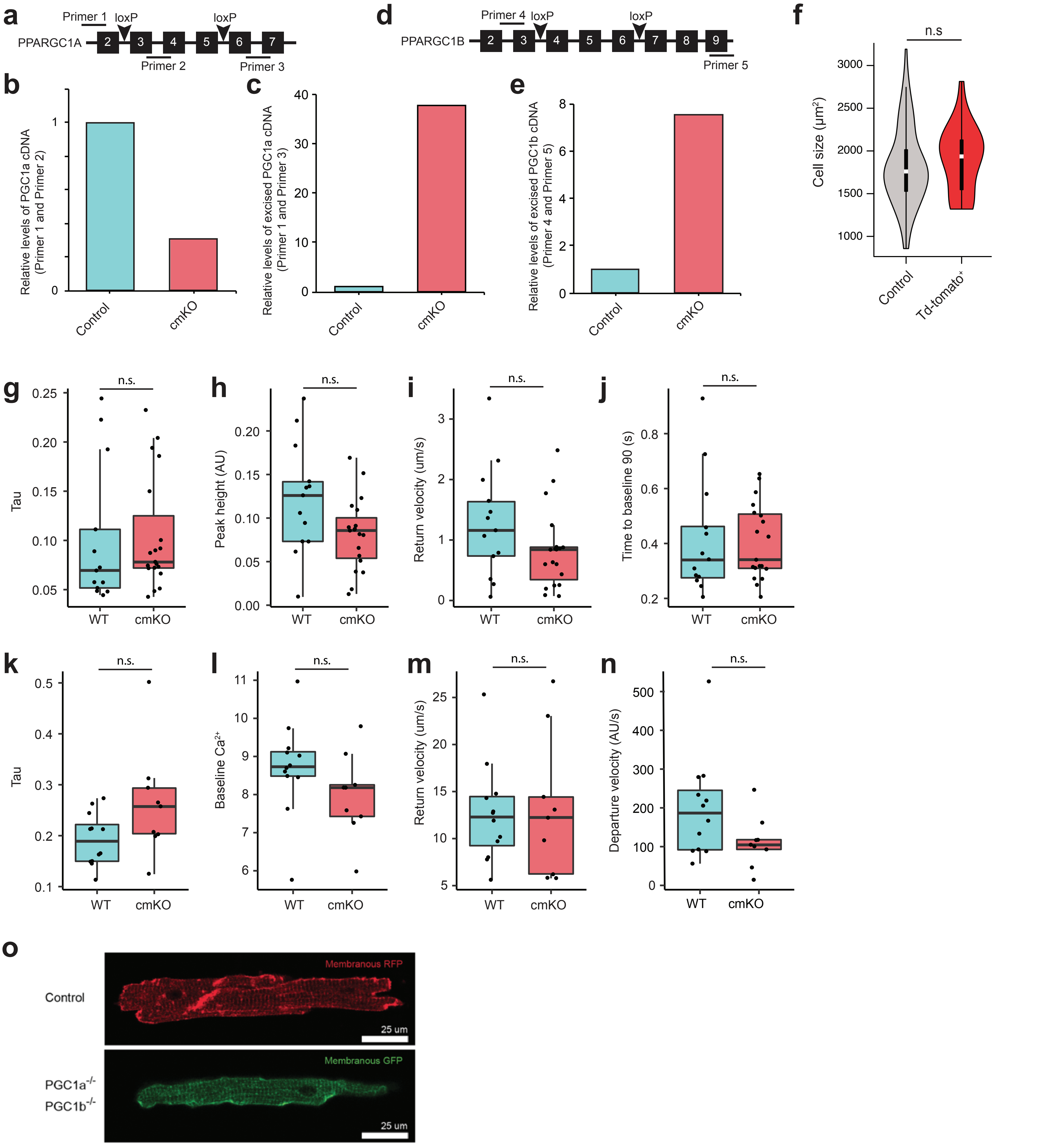

Fig. S2: PGC1 cmKO validation and additional functional metrics
a, Diagram showing exons 2–7 of PGC1α and the use of qPCR primers to quantify levels of PGC1α in control and cmKO CMs. **b**, Normalized levels of PGC1α in control (teal) and cmKO (red) CMs. **c**, Normalized levels of excised PGC1α. **d**, Diagram showing exons 2-9 of PGC1β and the use of qPCR primers to quantify levels of PGC1β. **e**, Normalized levels of excised PGC1β. f, Cell size measurements for myocytes isolated from p28 hearts of Ai9 mice injected with AAV9-cTnT-Cre at p0 (p-value=0.33). Control n=87, Td-tomato n=26 Student's t-test was used. **g–j**, Contractility parameters for control and PGC1 cmKO CMs. control n=13, cmKO n=19. (p-value=(g) 0.87, (h) 0.056, (i) 0.15 (j) 0.86) Student's t-test was used. **k–n**, Calcium transient parameters for control and PGC1 cmKO CMs. control n=12, cmKO n= 9. (p-value=(k) 0.094, (l) 0.166, (m) 0.89 (n) 0.062). AUs are arbitrary units. **o**, Representative images of control (membranous RFP) and PGC1-deficient (membranous GFP) P28 mouse CMs showing maintenance of t-tubule structure. scale bars = 25 µm. Student's t-test was used in panels g-j and Mann-Whitney-Wilcoxon test was used in panel n. Box-and-whiskers plot represents the maxima, 75th percentile, median, 25th percentile, and minima. N.S. p-value>0.05.

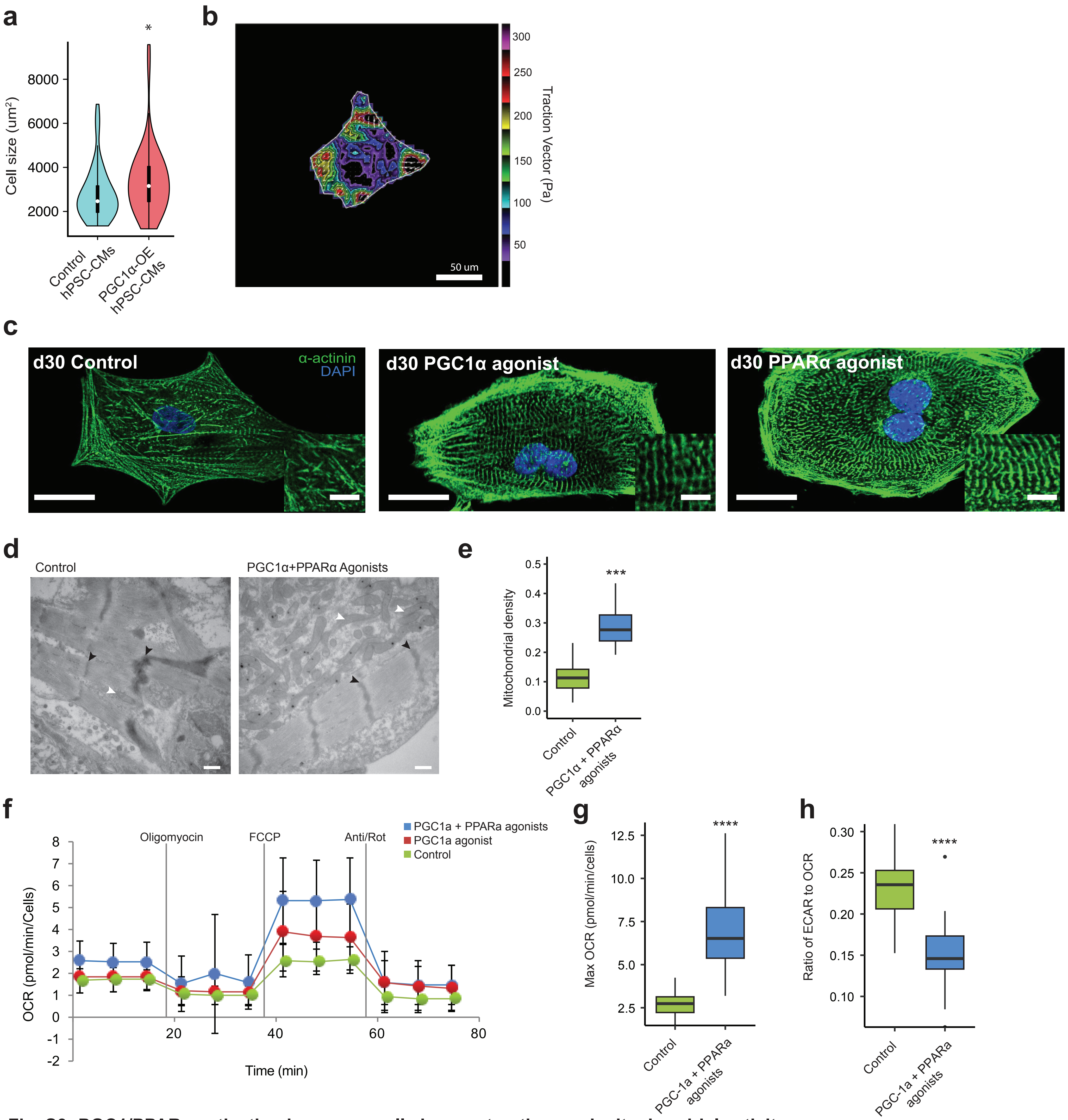

Fig. S3: PGC1/PPARα activation increases cell size, contraction, and mitochondrial activity
a, Cell area quantification of human PSC-CMs transfected with control (teal) and GFP-PGC1 (red) expression construct, measured 4 days after transfection (p-value=0.037). Student's t-test was used. Control n=55, GFP-PGC1 n=46. **b**, Fourier transform traction force microscopy showing force vectors during PSC-CM contraction. **c**, Images of day 30 human PSC-CMs treated with PGC1/PPARα agonists for 2 weeks after differentiation. **d**, Electron microscopy images of control and PGC1/PPARα agonist-treated day 30 PSC-CMs. Scale bar = 500 nm. White arrowheads show mitochondria and black arrowheads show z-bands. **e**, Mitochondrial density quantified from EM images (p-value =0.00017). Student's t-test was used. Control (green) n=9, Treated (blue) n=15
f, Seahorse XF96 measurements of oxygen consumption rate in control and agonist-treated d30 PSC-CMs. **g**, quantification of maximum oxygen consumption rate (p-value=1.4e-6) Control (green) n=17, Treated (blue) n=18 Student's t-test was used.
h. Ratio of ECAR to OCR (p-value=5.5e-6). Student's t-test was used. Control (green) n=17, Treated (blue) n=18. *>0.05, ***>0.001, ****>0.0001

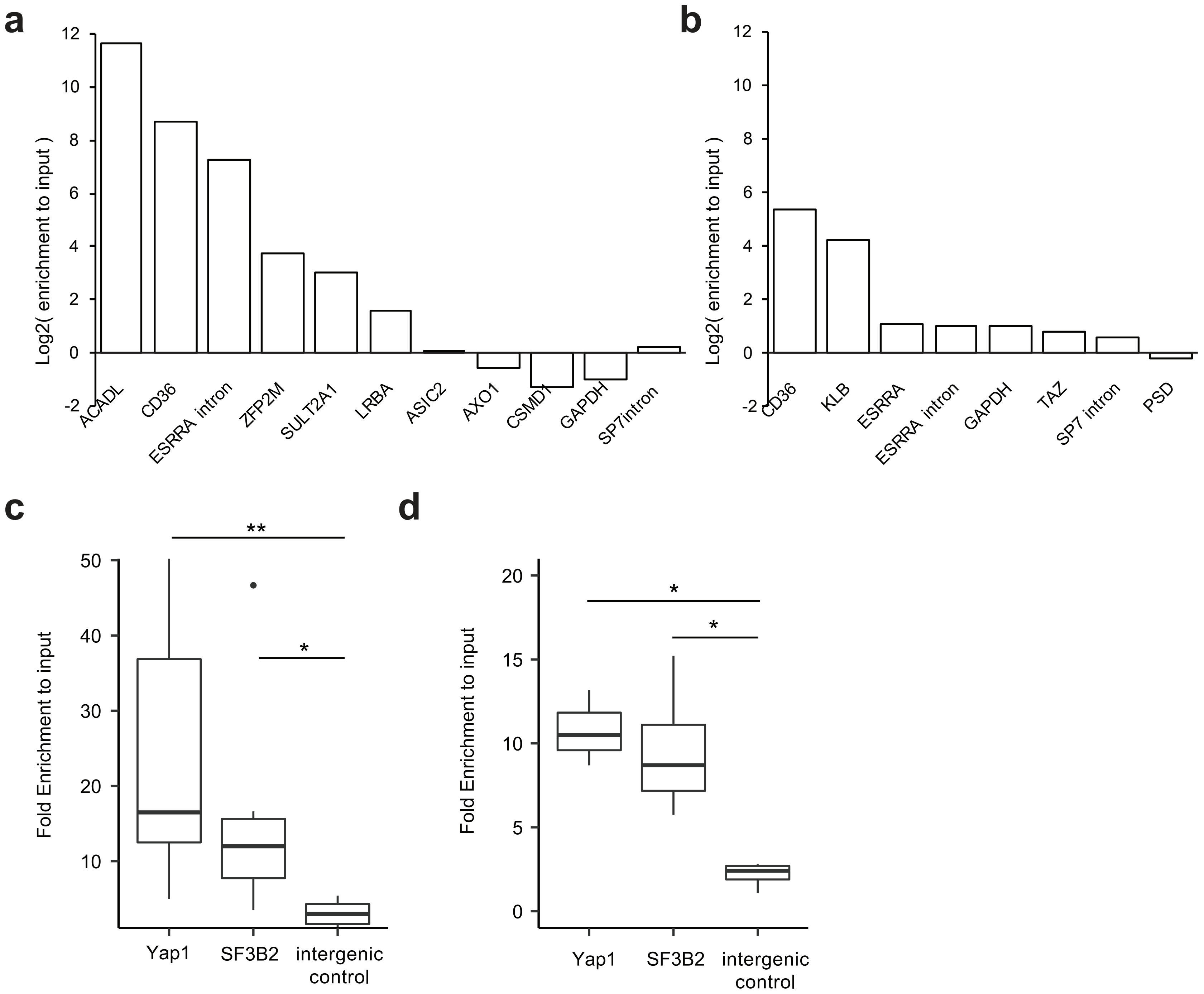

Fig. S4: PGC1/PPARα are physically associated with the promoters of Yap1/Sf3b2

a, b, Antibody validation for chromatin immunoprecipitation (ChIP). ChIP analysis showing enrichment of promoter sequences of known targets of PGC1/PPARα and control DNA regions (GAPDH, SP7 intron, ESRRA intron). **c, d**, PGC1/PPARα ChIP-qPCR analysis showing fold enrichment of Yap1 and SF3B2 promoter regions compared to an intergenic control (p-value= (c) 0.029, 0.029, (d) 0.0043, 0.015). (c) n=4, (d) n=6. Mann-Whitney-Wilcoxon test was used. p-value *<0.05, **<0.01

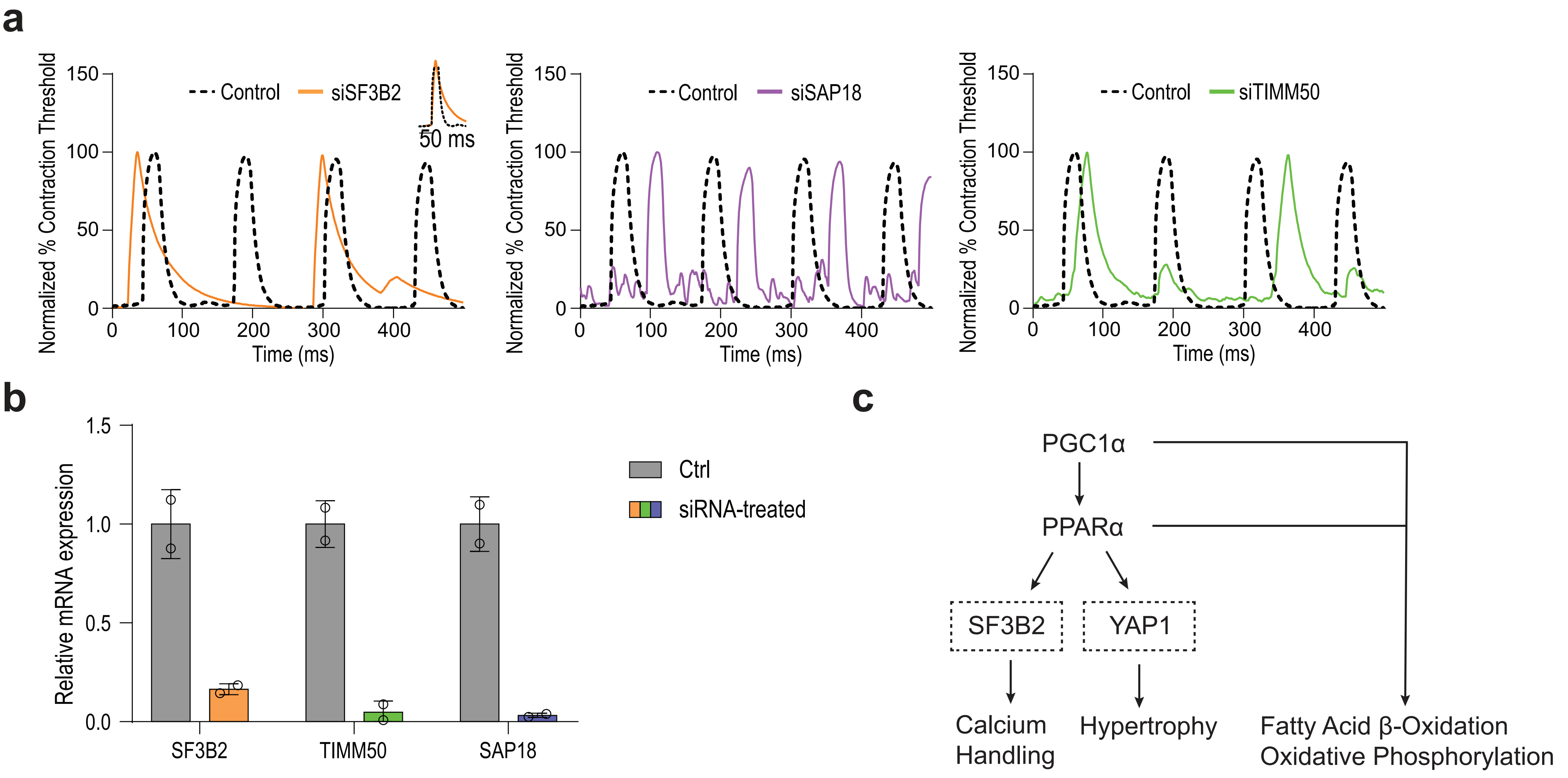

Fig. S5: Contractility analysis, siRNA efficiency, and working model
a, Effects of validated hits on contractility with traces from initial analysis shown by normalized percentage of contraction threshold. **b**, Knockdown efficiency of validated hits SF3B2 (orange), TIMM50 (green), and SAP18 (purple). n=2 **c**, Working model on postnatal CM maturation.
