# Supplementary material for: PGC1/PPAR drive cardiomyocyte maturation at single cell level via YAP1 and SF3B2
Source: Nat Commun. 2021 Mar 12;12:1648. doi: 10.1038/s41467-021-21957-z (PMC7955035; doi:10.1038/s41467-021-21957-z)
Supplement: Supplementary file 4 — Description of Additional Supplementary files [file 41467_2021_21957_MOESM4_ESM.docx]

Description of additional supplementary infomration

Title: Dataset 1

Description: Primer sequence information
