# Supplementary material for: PGC1/PPAR drive cardiomyocyte maturation at single cell level via YAP1 and SF3B2
Source: Nat Commun. 2021 Mar 12;12:1648. doi: 10.1038/s41467-021-21957-z (PMC7955035; doi:10.1038/s41467-021-21957-z)
Supplement: Supplementary file 5 — Reporting Summary [file 41467_2021_21957_MOESM5_ESM.pdf]

nature research

Corresponding author(s): Chulan Kwon

Last updated by author(s): 2-1-2021

Reporting Summary

Nature Research wishes to improve the reproducibility of the work that we publish. This form provides structure for consistency and transparency in reporting. For further information on Nature Research policies, see Authors & Referees and the Editorial Policy Checklist.

Statistics

For all statistical analyses, confirm that the following items are present in the figure legend, table legend, main text, or Methods section.

n/a	Confirmed	
☐	☒	The exact sample size (n) for each experimental group/condition, given as a discrete number and unit of measurement
☐	☒	A statement on whether measurements were taken from distinct samples or whether the same sample was measured repeatedly
☐	☒	The statistical test(s) used AND whether they are one- or two-sided<br>*Only common tests should be described solely by name; describe more complex techniques in the Methods section.*
☒	☐	A description of all covariates tested
☐	☒	A description of any assumptions or corrections, such as tests of normality and adjustment for multiple comparisons
☐	☒	A full description of the statistical parameters including central tendency (e.g. means) or other basic estimates (e.g. regression coefficient) AND variation (e.g. standard deviation) or associated estimates of uncertainty (e.g. confidence intervals)
☐	☒	For null hypothesis testing, the test statistic (e.g. F, t, r) with confidence intervals, effect sizes, degrees of freedom and P value noted<br>*Give P values as exact values whenever suitable.*
☒	☐	For Bayesian analysis, information on the choice of priors and Markov chain Monte Carlo settings
☒	☐	For hierarchical and complex designs, identification of the appropriate level for tests and full reporting of outcomes
☒	☐	Estimates of effect sizes (e.g. Cohen's d, Pearson's r), indicating how they were calculated

Our web collection on statistics for biologists contains articles on many of the points above.

Software and code

Policy information about availability of computer code

Data collection	MetaXpress (6), IonWizard (7.2), ImageJ (1.8.0), Seahorse Xfe96 Analyzer Wave (2.6), zUMIs (2.5.0), STAR (2.7), and Leica Application Suite (X) were used for data collection.
Data analysis	Code used for data analysis can be found at https://github.com/smurph50/Cardiomyocyte-maturation-scRNAseq

For manuscripts utilizing custom algorithms or software that are central to the research but not yet described in published literature, software must be made available to editors/reviewers. We strongly encourage code deposition in a community repository (e.g. GitHub). See the Nature Research guidelines for submitting code & software for further information.

Data

Policy information about availability of data

All manuscripts must include a data availability statement. This statement should provide the following information, where applicable:

- Accession codes, unique identifiers, or web links for publicly available datasets
- A list of figures that have associated raw data
- A description of any restrictions on data availability

Single cell RNA-sequencing data used in this study is available at the Gene Expression Omnibus GSE165917. Bulk RNA-seq can be found at GSE64403, GSE47948, GSE95762, GSE79883.

Field-specific reporting

Please select the one below that is the best fit for your research. If you are not sure, read the appropriate sections before making your selection.

☒ Life sciences ☐ Behavioural & social sciences ☐ Ecological, evolutionary & environmental sciences

For a reference copy of the document with all sections, see nature.com/documents/nr-reporting-summary-flat.pdf

Life sciences study design

All studies must disclose on these points even when the disclosure is negative.

Sample size	Power Analysis using an estimated effect size was used. For animal studies, we sought to reduce the number of animals used according to our approved animal handling protocol. Power Analysis was also used for animal studies.
Data exclusions	Single cell RNA-seq data were excluded if cells did not meet quality control measures.
Replication	Data from stem cell-derived cardiomyocytes was collected across multiple differentiations. Mice across multiple litters were used and from different parents. Effects of PGC1/PPAR agonists on PSC-CMs were performed across multiple labs at different institutions with successful replication.
Randomization	Samples and animals were randomly assigned to treatment groups.
Blinding	Co-authors that performed ImageJ analysis of cell size and cardiomyocyte function studies were blinded. Other experiments did not require blinding as data analysis was automated.

Reporting for specific materials, systems and methods

We require information from authors about some types of materials, experimental systems and methods used in many studies. Here, indicate whether each material, system or method listed is relevant to your study. If you are not sure if a list item applies to your research, read the appropriate section before selecting a response.

Materials & experimental systems

n/a	Involved in the study	
☐	☒	Antibodies
☐	☒	Eukaryotic cell lines
☒	☐	Palaeontology
☐	☒	Animals and other organisms
☒	☐	Human research participants
☒	☐	Clinical data

Methods

n/a	Involved in the study	
☒	☐	ChIP-seq
☒	☐	Flow cytometry
☒	☐	MRI-based neuroimaging

Antibodies

Antibodies used	PGC1a mouse monoclonal antibody (sc-518025) from Santa Cruz Biotechnology, PPARa ChIP-grade rabbit polyclonal antibody from Abcam (ab227074), Alpha actinin rabbit monoclonal [EP2527Y] from Abcam (ab68194)
Validation	ChIP-qPCR antibodies were validated by quantifying pulldown enrichment over input of known direct targets from the literature. This validation data is presented in the text of the manuscript and supplementary figures. The PPARa antibody has been certified by Abcam as ChIP-grade. The PGC1a antibody has been cited by 31 papers according to the manufacturer. The alpha actinin antibody was used to visualize sarcomere structures and matched expected distribution. The manufacturer lists references of this antibody being used to stain cardiomyocytes.

Eukaryotic cell lines

Policy information about cell lines

Cell line source(s)	E14 mouse embryonic stem cells and H9 human embryonic stem cells from WiCell. Both parents gave consent to the use of the embryo for research use for these cell lines.
Authentication	No authentication was used.
Mycoplasma contamination	Cell lines were not tested for mycoplasma contamination.

Commonly misidentified lines (See ICLAC register)	No commonly misidentified lines were used in this study.

Animals and other organisms

Policy information about studies involving animals; ARRIVE guidelines recommended for reporting animal research

Laboratory animals	Postnatal mice age P0 to P33 were used in this study. Mice were obtained from the Jackson Laboratory. PGC1a/b flox, Ai9, and Yap1flox mice were used. Male and female mice were used for all strains.
Wild animals	This study did not involve wild animals.
Field-collected samples	This study did not use field-collected samples.
Ethics oversight	Protocols for this study were approved by the animal care and use committee of Johns Hopkins Medical Institutions. We followed US NIH guidelines.

Note that full information on the approval of the study protocol must also be provided in the manuscript.
